# miR-100 maintains phenotype of tumor-associated macrophages by targeting mTOR to promote tumor metastasis via Stat5a/IL-1ra pathway in mouse breast cancer

**DOI:** 10.1038/s41389-018-0106-y

**Published:** 2018-12-19

**Authors:** Wei Wang, Yan Liu, Jian Guo, Huiwen He, Xue Mi, Chong Chen, Junling Xie, Shengnan Wang, Peng Wu, Fengqi Cao, Lipeng Bai, Qin Si, Rong Xiang, Yunping Luo

**Affiliations:** 1 Department of Immunology, Institute of Basic Medical Sciences, Chinese Academy of Medical Sciences and Peking Union Medical College, 100005 Beijing, China; 2 Collaborative Innovation Center for Biotherapy, School of Basic Medical Sciences, Chinese Academy of Medical Sciences and Peking Union Medical College, 100005 Beijing, China; 3 Department of Immunology, Medical School of Nankai University, 300071 Tianjin, China

## Abstract

Tumor-associated macrophages (TAMs), the main part of immune cells in tumor microenvironment (TME), play a potent role in promoting tumorigenesis through mechanisms such as stimulating angiogenesis, enhancing tumor migration and suppressing antitumor immunity. MicroRNAs (miRNAs) are considered as crucial regulators in multiple biological processes. The relationship between miRNAs and macrophages function has been extensively reported, but the roles that miRNAs play in regulating TAMs phenotype remain unclear. In this study, we screened highly expressed microRNAs in TAMs, and first identified that miR-100 represented a TAMs-high expression pattern and maintained TAMs phenotype by targeting mTOR signaling pathway. Moreover, miR-100 expression level in TAMs was positively related to IL-1ra secretion, a traditional immune-suppressive cytokine, which was determined to promote tumor cells stemness via stimulating Hedgehog pathway. Mechanism study suggested that mTOR/Stat5a pathway was involved in IL-1ra transcriptional regulation process mediated by miR-100. More importantly, tumor metastasis and invasion capacity were significantly decreased in a 4T1 mouse breast cancer model injected intratumorally with miR-100 antagomir, and combination therapy with cisplatin showed much better benefit. In this study, we confirm that highly expressed miR-100 maintains the phenotype of TAMs and promotes tumor metastasis via enhancing IL-1ra secretion. Interfering miR-100 expression of TAMs in mouse breast cancer model could inhibit TAMs pro-tumor function and reduce tumor metastasis, which suggests that miR-100 could serve as a potential therapy target to remodel tumor microenvironment in breast cancer.

## Introduction

Tumor-associated macrophages (TAMs), one of the key components of tumor microenvironment (TME), promote tumor progression in various aspects including tumor cell growth, survival, invasion, metastasis, angiogenesis, inflammation, and immunoregulation^1^. Physiologically, macrophages are categorized into two different phenotypes. M1, or classically activated macrophages, induced by Lipopolysaccharide (LPS) and/or gamma-interferon, are capable of secreting inflammatory cytokines such as TNF-alpha and IL-12 and thus eradicating invasive microbes or tumor cells. M2, or alternatively activated macrophages, are induced by IL-4/IL-13 or IL-10 and acquire an anti-inflammatory phenotype characterized by secreting IL-10 and TGF-beta^2^. Growing evidences show that TAMs represent a M2-like phenotype and closely correlate with clinical prognosis of breast cancer, non-small cell lung cancer, and ovarian cancer^3^. Besides, TAMs are proven to support tumor progression by promoting survival^4^, migration and invasion^5^, epithelial–mesenchymal transition, and metastasis^6^ and they also play a key part in suppressing antitumor immunity^7^. These findings enable TAMs to be a promising target of cancer therapy^8^. Strategies about “re-educating” or eliminating tumor-promoting TAMs are undergoing preclinical or clinical evaluation^9,10^. As the concept of cancer stem cells (CSCs) has been widely recognized, TAMs are also gaining its popularity by strengthening cancer cell stemness property^11^. Although TAMs have been intensively studied in recent years, the mechanisms that regulate their phenotypes and function still largely remain unclear.

MicroRNAs (miRNAs), that are endogenous, approximately 23-nucleotide-long RNAs, play important roles by targeting mRNAs to direct their posttranscriptional repression both in animals and plants, establishing a new paradigm of gene regulation^12^. Highly conserved among vertebrates, miRNAs are considered to be involved in diverse physiological processes including cell proliferation, apoptosis, differentiation, and metabolism^13^. These miRNAs have also been proven to play important parts in many pathological situations, especially in cancer, where they could function as either oncogenes or tumor suppressors^14^. Expression profiles of miRNAs varies in different types of cancer as well as in different disease stages^15^. As oncogenes, miRNAs can accelerate tumor cells proliferation or enhance their stemness and metastasis ability by targeting tumor suppressors^16,17^. On the contrary, some miRNAs can suppress tumor growth or stemness by conducting their posttranscriptional regulation^18,19^.

miRNAs have also been recognized to regulate the phenotype and function of macrophages^20^. For instance, MiR-155, upregulated by NF-κB pathway, could promote the secretion of TNF-alpha and then boost inflammatory response^21^, while miR-164 could antagonize inflammation by targeting IRAK1 and TRAF6^22^. When it came to TAMs, miR-511-3p was the first reported miRNA, which could limit TAMs’ tumor-promoting functions and inhibit tumor growth^23^. We previously reported that miR-19a-3p could downregulate the M2 phenotype of TAMs by targeting Fra-1 and thus suppressing breast cancer progression^24,25^. Both TAMs and miRNAs have been intensively researched; however, the specific roles that miRNAs play in TAMs are still unclear.

In this study, we performed miRNAs sequencing in TAMs isolated from mouse breast tumor tissue and screened TAMs-related miRNAs induced by TME according to the sequencing results. Then we identified miR-100 as a crucial player to maintain M2 phenotype of TAMs both in vitro and in vivo by targeting mTOR pathway and upregulating the secretion of IL-1ra that could enhance tumor cell stemness. We also proved that miR-100 antagomir alone or combined with cisplatin could significantly suppress lung metastasis in mouse breast cancer model. Our findings provide evidences and give insights about miRNAs’ regulating TAMs and their potential as promising therapeutic targets.

## Results

### miR-100 represents TAMs-high expression in mouse breast cancer model and human breast cancer patients

**Fig. 1:**
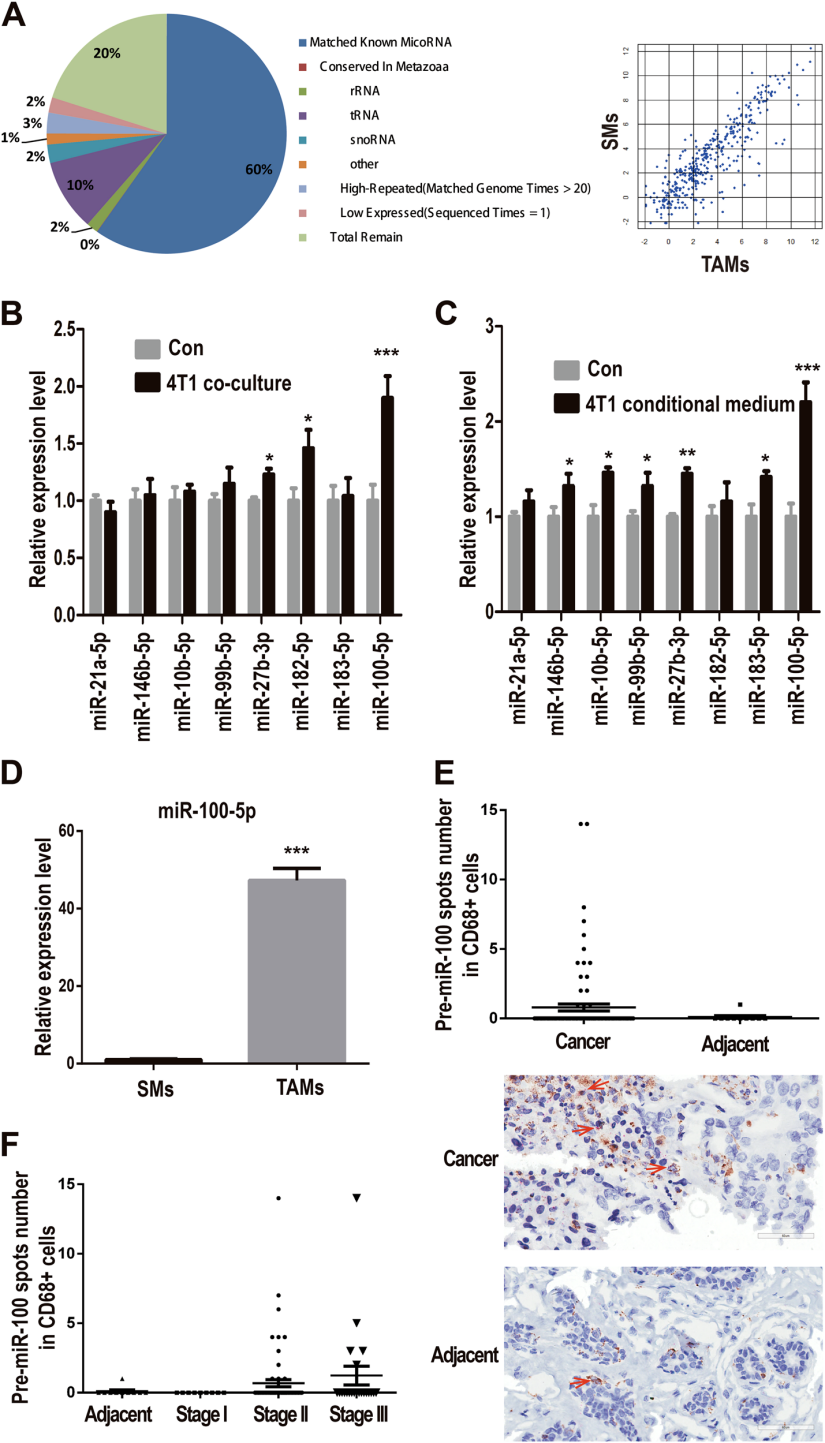
miR-100-5p is highly expressed in TAMs of mouse and human breast cancer. **a** Pie chart (left) for total reads distribution for categorized small RNAs in TAMs and pairwise comparison of known miRNAs expression between TAMs and SMs. Scatter plot (right) showed the log10 value of TPM, each plot represented one miRNA. **b**, **c** A panel of TAMs-high expressed miRNAs were detected by quantitative polymerase chain reaction (qPCR) in RAW264.7 cells, co-cultured with 4T1 mouse breast cancer cell (**b**) or 4T1 conditional medium (1640 medium: 4T1 supernatant = 1:1) (**c**). **d** The expression of miR-100-5p in TAMs was tested by qPCR. **e** The expression of pre-miR-100-5p was detected by RNAscope staining and the expression of macrophage marker CD68 was detected by IHC in human breast cancer tissue array, adjacent normal tissues as control samples. Numbers of pre-miR-100-5p spots (red arrow) in CD68+ cells from each cancer tissue or adjacent normal tissue were counted. Representative staining results were shown below. Scale bars, 60 µm. **f** Numbers of pre-miR-100-5p spots in CD68+ cells in different stages of breast cancer case were analyzed. Data were shown as mean ± s.e.m. from three independent experiments; ***P < 0.001, **P < 0.01, *P < 0.05

A syngeneic mouse breast cancer model was established with 4T1 mouse breast cancer cell line following the procedure in FigS. 1A. Mice were sacrificed when tumors grew up to 1000 mm^3^ and tumor tissues were harvested for TAMs isolation by magnetic beads. Macrophages from naïve mice spleen (SMs) served as control samples. After detecting the purity and vitality of the sorted cells by flow cytometry (FigS. 1B), TAMs and SMs’ RNA were extracted and miRNA Solexa sequencing was performed. 20M clean reads were mapped into pie-chart category. MiRNAs expression showed similar pattern between TAMs and SMs; however, a certain amount of TAMs-high expressed miRNAs was identified in different-expressed miRNAs pool (Fig. 1a). Moreover, highly expressed miRNAs (transcripts per million (TPM) >5000) that take up only less than 2% of total miRNAs occupied almost 80% of total reads. Therefore, eight candidate miRNAs were screened out with characteristics of both TAMs high-expression and TPM higher than 5000. Next, expression levels of these candidate miRNAs were, respectively, validated in macrophages co-cultured with 4T1 cells and macrophages cultured with 4T1 cells conditional medium. Among these candidates, miR-100 showed the most significant upregulation in TAMs (Fig. 1b, c), and the high expression of miR-100 was also validated in TAMs isolated from 4T1 mouse breast cancer tissues controlled by naïve mouse SMs (Fig. 1d). To validate miR-100 expression in clinical samples, signals of both precursor miR-100 (pre-miR-100) and macrophages marker CD68 were detected in human breast cancer tissue array simultaneously by RNAscope and immunohistochemical (IHC) staining. RNAscope is an approach for detecting pre-miRNAs in situ by using specifically designed probe and signal amplification method in the tissue array. It was found that pre-miR-100 expression in CD68^+^ macrophages was much higher in cancer tissues than that in the adjacent normal tissues (Fig. 1e). The expression of pre-miR-100 in total cells was also upregulated in cancer tissues compared with adjacent tissues (FigS. 1D).

Further, we processed the data according to different cancer stages and found an increased trend of pre-miR-100 expression in CD68^+^ cells along with cancer development (Fig. 1f). These results suggested that miR-100 was one of the highly expressed miRNAs of TAMs in breast cancer.

### miR-100 promotes the M2-polarization of macrophages and maintains TAMs phenotype both in vitro and in vivo

**Fig. 2:**
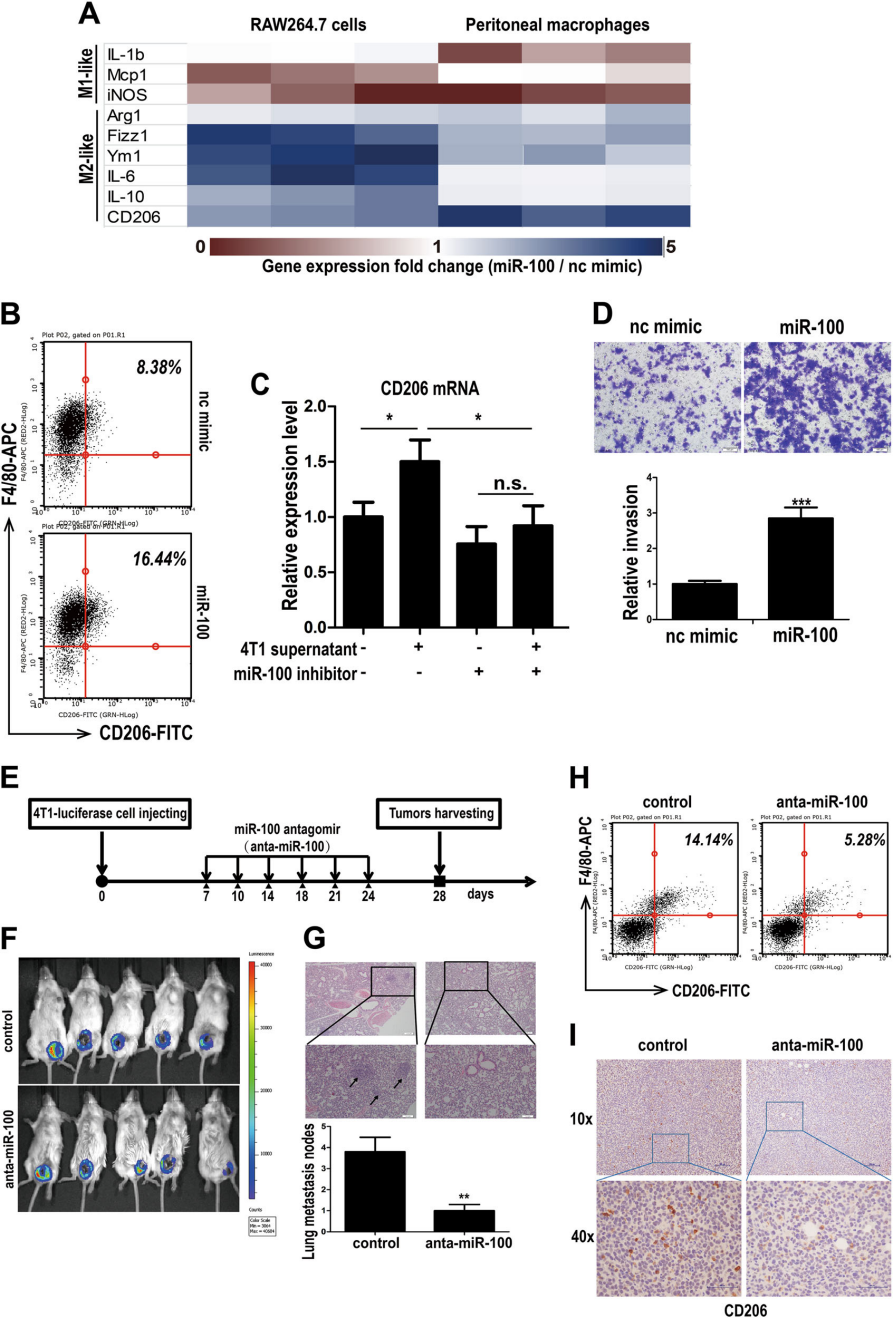
miR-100 promotes the M2-polorization of macrophages both in vitro and in vivo and enhances lung metastasis in 4T1 mouse breast model. **a** RAW 264.7 cells and mouse peritoneal macrophages (PMs) were transfected with miR-100 mimic for 24 h, nc mimic as negative control. Expression of M1 and M2 phenotype marker genes were detected by qPCR and expression foldchanges were shown in the hotmap from three independent experiments. **b** PMs were transfected with miR-100 mimic for 24 h, and the F4/80+ CD206+ population was measured by flow cytometry. **c** PMs were transfected with miR-100 inhibitor and cultured with or without 4T1 supernatant, and then CD206 mRNA expression was detected by qPCR. **d** Migration of 4T1 cells co-cultured with miR-100 mimic transfected RAW264.7 cells was detected by using transwell assay. Scale bars, 100 µm. **e** Flow chart of in vivo experiments. Luciferase-labeled 4T1 cells were inoculated in BALB/c mice, and then intratumorally injected miR-100 antigomir twice a week, nc antigomir as control, *n* = 5. **f** Primary tumor growth was detected by in vivo imaging after injection of luciferase substrate. **g** Numbers of lung metastasis nodes were counted and lung tissue HE staining pictures were shown. Scale bars, 100 µm. **h** Flow cytometry to detect the percentage of F4/80+ CD206+cells in tumor tissues of both groups. **i** IHC staining for CD206 expression in 4T1 mouse breast cancer tissue. Scale bars, 10 µm. Data was shown as mean ± s.e.m. from three independent experiments; ***P < 0.001, **P < 0.01, *P < 0.05

**Fig. 3:**
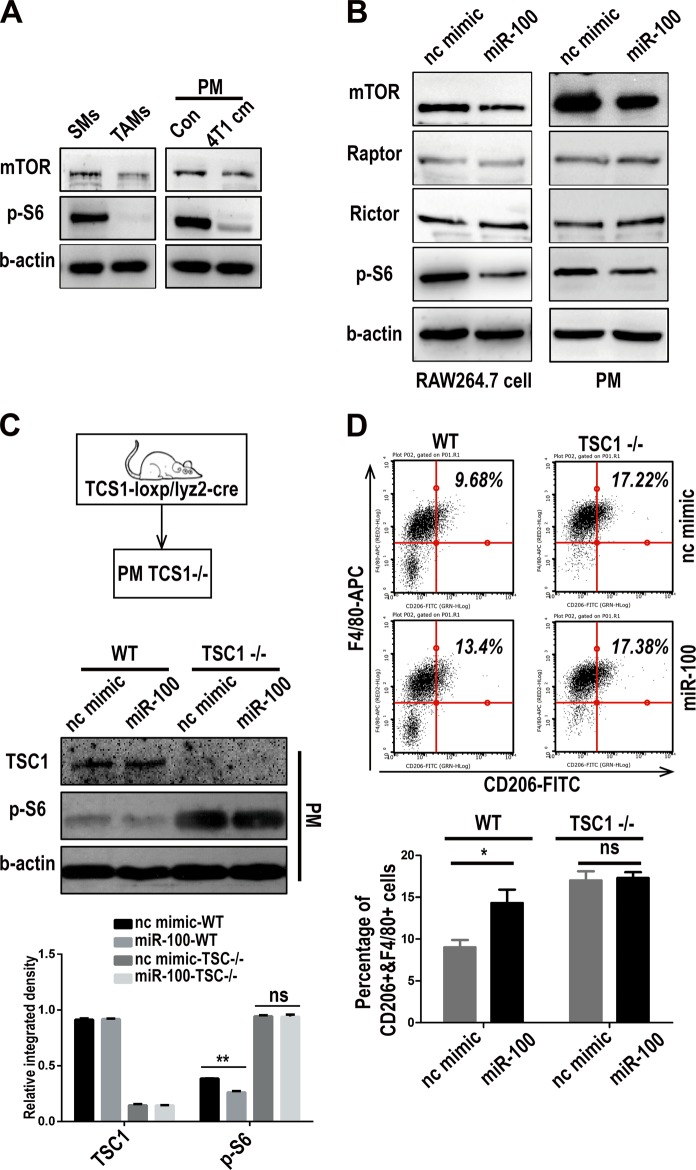
miR-100 maintains TAMs phenotype dependent on target-inhibiting mTOR pathway. **a** mTOR and phosho-S6 expression were detected by Western blot in TAMs/SMs and PMs cultured with 4T1 conditional medium. **b** Expression of mTOR pathway-related proteins was detected by Western blot in RAW264.7 cells and PMs that were transfected with miR-100 mimic, β-actin as a loading control. **c** Western blot to detect TSC1 and p-S6 expression in PMs isolated from TSC1 myeloid-specific knockout mouse and transfected with miR-100 mimic. Quantitation of the Western blot results is at the bottom. **d** Flow cytometry to detect the percentage of F4/80+CD206+ population in miR-100 mimic transfected PMs isolated from TSC1 myeloid-specific knockout mouse. Data are shown as mean ± s.e.m. from three independent experiments, *P < 0.05

To investigate whether miR-100 could regulate macrophage phenotype and function, miR-100 mimic was transfected into both RAW264.7 cells and mouse primary peritoneal macrophages (PMs), and then the expressions of M1 and M2 marker genes were detected by qPCR. In the heatmap of expression foldchange, M2 marker genes were significantly upregulated in miR-100 overexpressed (O/E) group, while M1 marker genes were downregulated, demonstrating that miR-100 could shift macrophages toward M2 polarization (Fig. 2a). Besides, CD206, one of the key markers of TAMs, was found to have a positive correlation with miR-100, as miR-100 O/E PMs showed an increased percentage of F4/80^+^CD206^+^ cells compared with that in the negative control group (Fig. 2b). Meanwhile, after knocking down miR-100 expression of macrophages, tumor cell supernatant failed to induce CD206 expression, which demonstrated that miR-100 was essential for TAMs phenotype formation induced by tumor cells (Fig. 2c). However, miR-100 did not seem to alter antigen-presenting molecules expression and phagocytosis ability of macrophages (FigS. 2A, 2B). Moreover, miR-100 O/E macrophages supernatant could significantly promote 4T1 cells invasion ability according to the transwell assay results (Fig. 2d). Then we explored the function of miR-100 in 4T1 mouse breast cancer model by knocking down miR-100 with its antagomir, especially in terms of macrophages phenotype and tumor metastasis. Mice were inoculated with luciferase-labeled 4T1 cells. After randomly grouping, miR-100 antagomir or negative control, a chemically modified miRNA inhibitor, was injected intratumorally twice a week (Fig. 2e). Primary tumor and lung tissues were harvested at 28 days post inoculation for further TAMs phenotype analysis. There was no change in body weight between two groups (FigS. 3A), and qPCR result showed that miR-100 was knockdown efficiently in TAMs isolated by magnetic beads (FigS. 3B). In vivo imaging was performed to observe primary tumor development (Fig. 2f). Tumor volume and tumor weight also showed no significant changes (FigS. 3C and 5D). However, lung tissue HE staining indicated that miR-100 antagomir injection could significantly attenuate tumor metastasis capacity (Fig. 2g), and lung/body weight ratio result proved it as well (FigS. 3E). When further detecting TAMs phenotype, there was a significant decrease of F4/80^+^CD206^+^ cells subpopulation in mice tumor tissue after injecting miR-100 antagomir compared with the negative control (Fig. 2h). Consistent with these data, a significant downregulated CD206 expression in miR-100 antagomir group could be observed by immunohistochemical staining (Fig. 2i), and CD206 mRNA expression level showed the same pattern (FigS. 3F). These results indicated that miR-100 could promote TAMs phenotype and function both in vitro and in vivo.

### miR-100 maintains TAMs phenotype dependent on target-inhibiting mTOR pathway

**Fig. 4:**
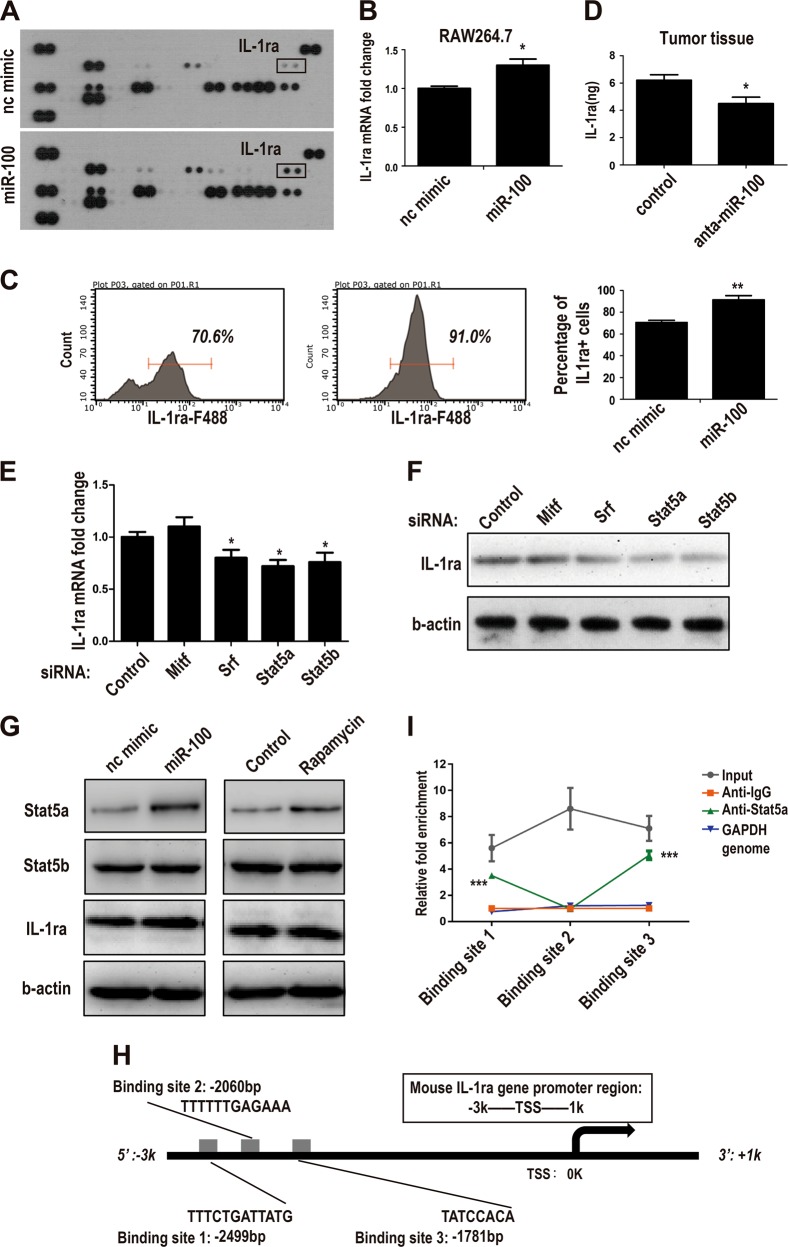
miR-100 increases IL-1ra secretion through stat5a-mediated transcriptional regulation. **a** Supernatants of RAW264.7 cells transfected with miR-100 mimic and nc mimic were collected and secreted cytokines were measured by the mouse cytokine array panel. **b** IL-1ra mRNA expression was measured by qPCR in miR-100 mimic transfected RAW264.7 cells. **c** IL-1ra+ cells percentage in PMs transfected with miR-100 mimic were measured by flow cytometry. **d** IL-1ra expression in miR-100 antagomir-treated 4T1 mouse breast cancer tissues was detected by ELISA. **e**, **f** qPCR and Western blot to detect IL-1ra expression in RAW264.7 cells transfected with siRNAs targeting several candidate TFs. **g** Western blot to detect Stat5a and Stat5b expression in RAW264.7 cells transfected with miR-100 mimic or treated with rapamycin. **h** Stat5a binding sites were predicted by ALLGEN database in IL-1ra promoter region from −3k to +1k. **i** Three stat5a binding sites were validated by qPCR in genomic DNA extracted from RAW264.7 cells. Data were shown as mean ± s.e.m. from three independent experiments; **P < 0.01, *P < 0.05

Published data proved that mTOR was the direct target gene of miR-100 in tumor cells^26^. Whether mTOR was also the target gene of miR-100 in macrophages was then investigated. At first, the expression of mTOR and signal pathway related proteins in primary TAMs and PMs were detected. mTOR and its downstream molecule activity (p-S6) were downregulated in TAMs controlled by SMs, and PMs that were cultured with 4T1 conditional medium (Fig. 3a, quantitation in FigS. 4A). To further validate the relationship between miR-100 and mTOR signal pathway in macrophages, miR-100 was overexpressed either in RAW264.7 cells or PMs. The results showed a significantly decreased expression of mTOR and its downstream related molecules in miR-100 highly expressed RAW264.7 cells and PMs (Fig. 3b, quantitation in FigS. 4B). All these results indicated that mTOR signal pathway blockage in TAMs was due to high expression of miR-100. A genetic-knockout mouse model was used for further validation of this mechanism. PMs were isolated from TSC1 (mTOR suppressor) gene myeloid-specific knockout mice (TSC-loxp/lyz2-cre), in which mTOR pathway was spontaneously activated. Western blot results with quantitation below showed that miR-100 could repress p-S6 expression in wildtype PMs but did not work in TSC1^−/−^ ones (Fig. 3c). Meanwhile, flow cytometry assay showed that miR-100 could still upregulate CD206 expression in wildtype PMs, whereas it had no influence under mTOR activated status (Fig. 3d). mRNA detection of CD206 showed the same pattern (FigS. 4C). All above data supported that miR-100 maintained TAMs phenotype in an mTOR pathway-dependent way.

### miR-100 increases IL-1ra secretion through stat5a-mediated transcriptional regulation

**Fig. 5:**
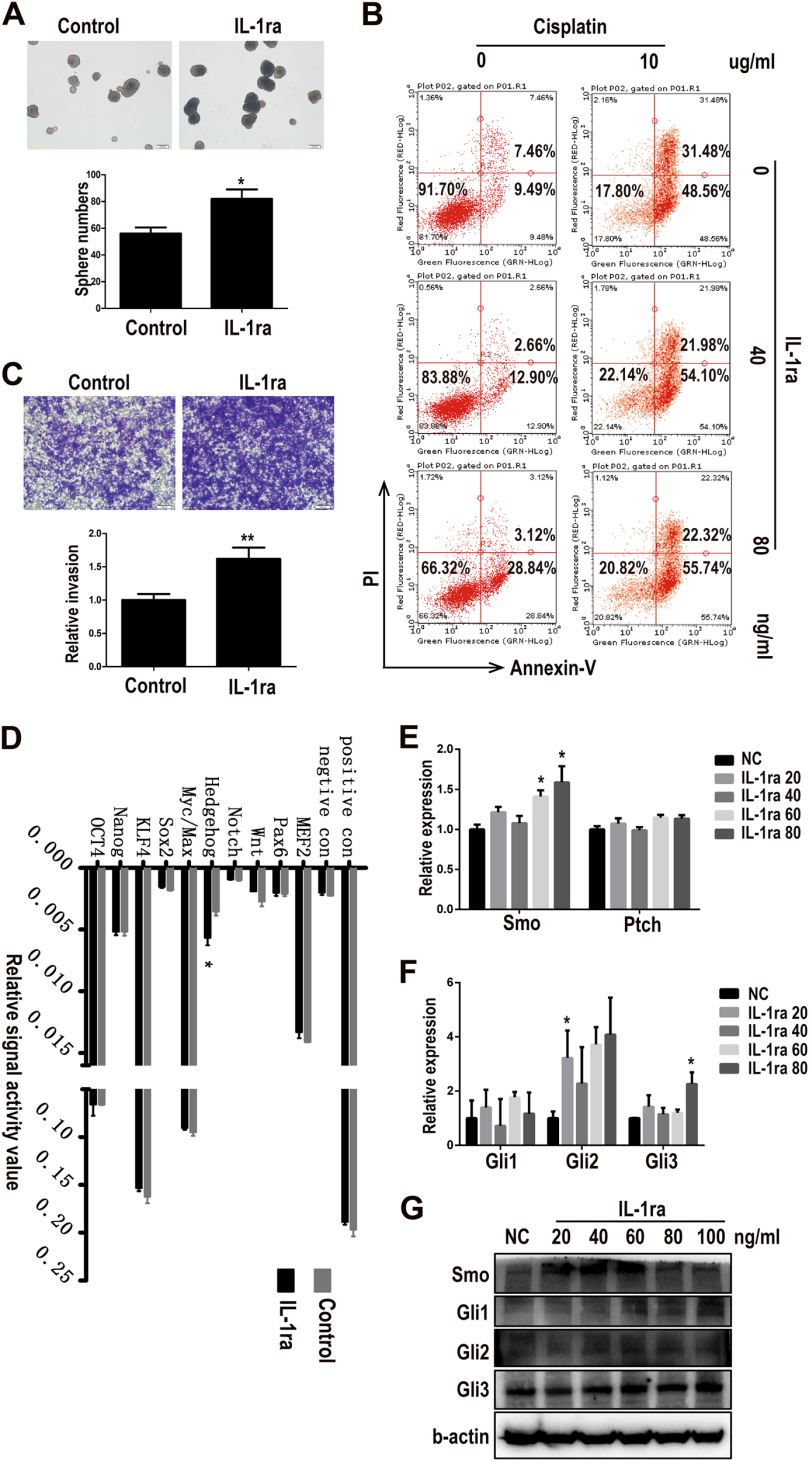
IL-1ra enhances tumor cell stemness and chemo-resistant ability via activating Hedgehog pathway. **a** 4T1 cells were cultured in sphere formation medium with or without IL-1ra (20 ng/mL) for 7 days, and the numbers of spheres were counted under a microscope. **b** Drug resistance ability to cisplatin of 4T1 cells was measured by flow cytometry. PI+ Annexin V+ represents late apoptosis status and PI−Annexin− represents living status. **c** Migration ability of 4T1 cells treated with IL-1ra was measured using transwell assay. Scale bars, 100 µm. **d** Stemness signal pathway array for IL-1ra-treated 4T1 cells. **e** Expressions of receptor(Smo) and inhibitor(Ptch) of Hedgehog pathway in IL-1ra-treated PMs were detected by qPCR. **f** Expressions of Hedgehog pathway transcript factors in IL-1ra-treated PMs were detected by qPCR. **g** Expressions of Hedgehog pathway molecules in IL-1ra-treated PMs were detected by Western blot. Data were shown as mean ± s.e.m. from three independent experiments; **P < 0.01, *P < 0.05

To inspect overall functional changes of macrophages induced by miR-100, a cytokine array for miR-100 O/E macrophages was performed. Among all detected cytokines, IL-1ra represented the most significant foldchange (Fig. 4a). IL-1ra upregulation of the array result was validated respectively in RNA (Fig. 4b) and protein level (Fig. 4c), indicating that this kind of secretion-increasing may rely on transcriptional regulation. Moreover, ELISA detection of supernatant from 4T1 mouse breast cancer tissues showed that miR-100 antagomir could inhibit IL-1ra production by macrophages (Fig. 4d). To explore the mechanisms in detail, two transcriptional factors (TFs) pools were merged to generate a candidate TFs pool involved in miR-100 and IL-1ra pathway. One pool was mTOR pathway negatively related TFs (mRNA-seq data online), the other was TFs that have potential to bind to IL-1ra promoter predicted by ALLGEN database (FigS. 5A). After setting a threshold, six TFs were listed (FigS. 5B), and only four of them could be expressed by macrophages. We used siRNA to knock down these TFs expression (FigS. 5C), then checked mRNA and protein expression of IL-1ra. Only Si-Stat5a and Si-Stat5b could downregulate IL-1ra at both transcriptional (Fig. 4e) and protein levels (Fig. 4f, quantitation in FigS. 5D). To clarify the specific regulation relationship, miR-100 mimic was used to repress mTOR pathway, and then Stat5a and Stat5b expressions were detected; rapamycin treatment used as a positive control. The results showed that Stat5a represented a negative correlation with mTOR pathway activation and could be mediated by miR-100 (Fig. 4g, quantitation in FigS. 5E), which suggested Stat5a was the key TF involved in the transcriptional regulation. A ChIP-PCR assay was performed to validate Stat5a binding situation with three putative binding sites predicted by ALLGEN (Fig. 4h). Based on the qPCR result, binding sites 1 and 3 could certainly bind with Stat5a protein (Fig. 4i). The data above supported that Stat5a was the key TF involved in the miR-100/mTOR/IL-1ra regulation axis.

### IL-1ra enhances tumor cell stemness and chemo-resistant ability via activating Hedgehog pathway

**Fig. 6:**
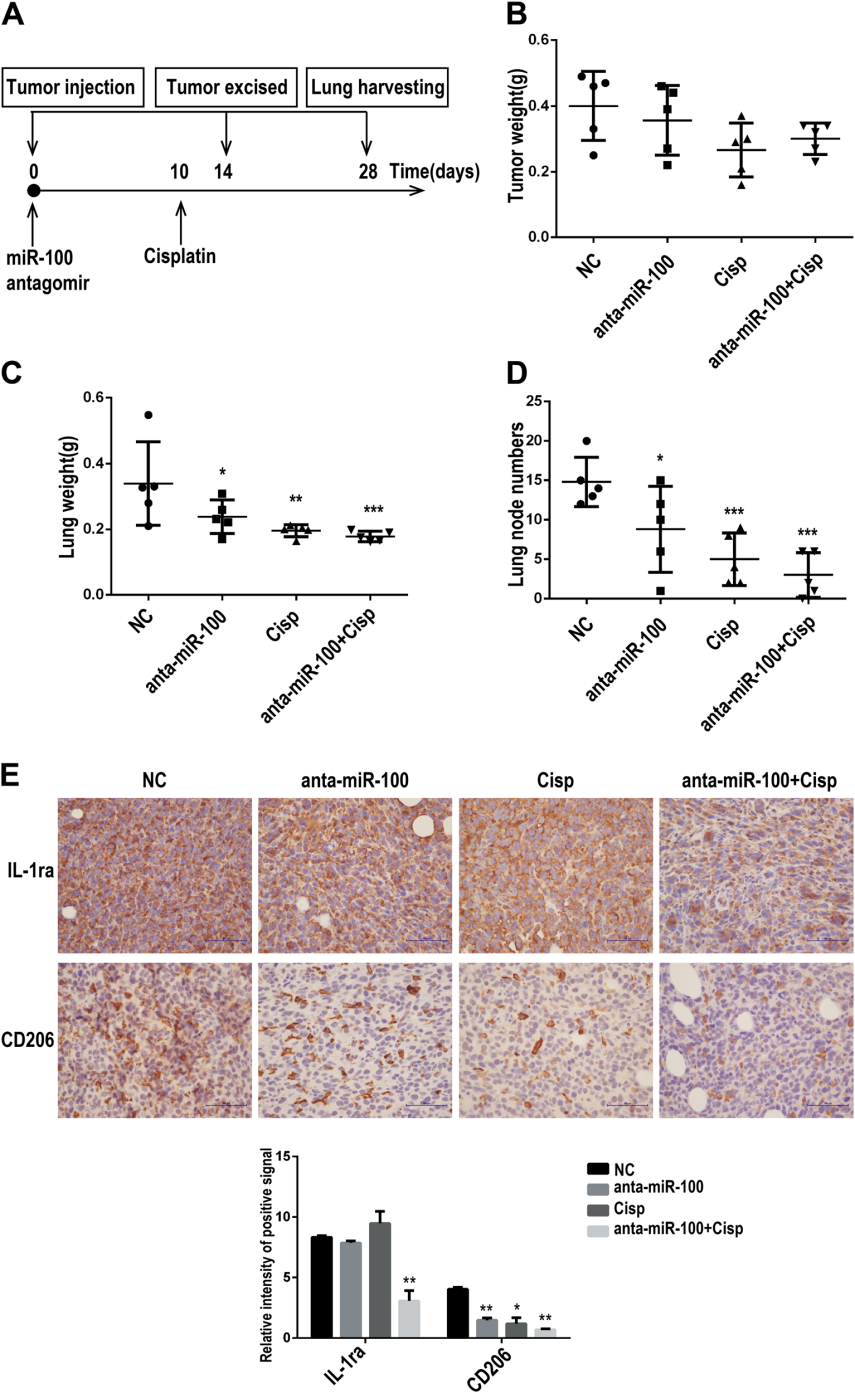
MiR-100 antagonist inhibits lung metastasis and enhances chemotherapy -sensitivity of tumor cells in mouse breast cancer model. **a** Scheme of miR-100 knocking-down and cisplatin combination therapy in 4T1 mouse breast cancer model. Eight-week-old female mice were randomly distributed into four groups (NC, anta-miR-100, cisplatin, anta-miR-100+cisplatin). **b** Tumor weights of all the groups were recorded on day 14. **c** Lung weights of all mice were measured on day 28. **d** Lung metastatic nodes were counted. **e** Expression of IL-1ra and CD206 was detected by IHC in tumor tissues and the intensity of the positive signal was analyzed by Aperio ImageScope. Scale bars, 100 µm; *n* = 5, ***P < 0.001, **P < 0.01, *P < 0.05

Given the fact that IL-1ra was secreted more as miR-100 was highly expressed, the next step was to investigate the role that IL-1ra played in TME, especially in regulating tumor cells function. Recombinant IL-1ra protein was used for treating 4T1 tumor cells. We first checked the tumor cell sphere formation ability, a widely recognized assay to detect cancer cell stemness property, after IL-1ra treatment, which was enhanced when adding IL-1ra (Fig. 5a). Then, either drug resistance ability to cisplatin or invasion of tumor cells was detected. The data demonstrated that IL-1ra could improve drug resistance ability to cisplatin (Fig. 5b) and promote invasion of 4T1 cells (Fig. 5c). These findings suggested that IL-1ra could enhance the tumor cell stemness property. To further develop the mechanisms underlying this phenomenon, we screened ten tumor cell stemness-related signal pathways. Only Hedgehog signal pathway was stimulated significantly by IL-1ra, while the others did not show any obvious changes (Fig. 5d). Then the expression of Hedgehog pathway receptor Smo (GPCR-like protein Smoothened) and inhibitor Ptch (Patched Homolog 1) were detected by qPCR in 4T1 cells treated with different concentration of IL-1ra. Results showed that Smo was upregulated by IL-1ra while Ptch stayed unaffected (Fig. 5e). Hedgehog pathway transcript factors were also tested by qPCR and it was found that Glioma-associated oncogene homolog 2 (Gli2) and Gli3 were upregulated by IL-1ra (Fig. 5f). Protein level validation showed that Smo was also upregulated along with Gli1 and Gli2 (Fig. 5g, quantitation in FigS. 6A). These results suggested that miR-100 may upregulate IL-1ra expression via activating Hedgehog pathway.

### miR-100 antagonist inhibits lung metastasis and enhances chemotherapy-sensitivity of tumor cells in mouse breast cancer model

Data in Figs. 2g and 5b have showed that inhibition of miR-100 could suppress lung metastasis in 4T1 mouse breast cancer model and IL-1ra, upregulated by miR-100 overexpression, could enhance 4T1 cells resistance to cisplatin. Then we designed a chemotherapy combined administration for 4T1 mouse breast cancer, to explore the potential of miR-100 clinical application (Fig. 6a). Drug administration methods are listed in FigS. 6B. Primary tumors were sectioned and weighed at day 14 post inoculation; the mice were continued to be dosed and then observed lung metastasis on day 28. There was no significant change in terms of primary tumor growth (FigS. 6C) and tumor weight (Fig. 6b). However, lung metastasis of three therapeutic groups was significantly inhibited, and lung metastasis in combined administration of miR-100 antagomir and cisplatin group was the most significantly inhibited than either monotherapy group (Fig. 6c, d; FigS. 6D). Immunohistochemical staining of tumor tissues showed that IL-1ra and CD206 were downregulated in all three treatment groups and showed the least expression of both molecules in combination therapy group (Fig. 6e).

**Fig. 7:**
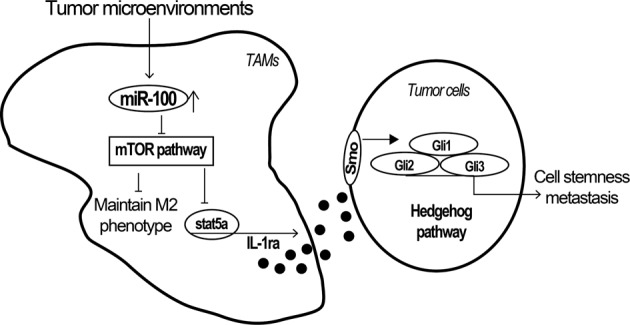
The work model of miR-100-maintained TAMs phenotype in TME. MiR-100 is highly -expressed in TAMs and overexpression of miR-100 can promote TAMs phenotype by regulating mTOR signaling pathway and upregulate IL-1ra secretion via Stat5a transcriptional regulation, which could enhance the stemness and migration property of 4T1 tumor cells through activating Hedgehog signaling pathway. Inhibition of miR-100 could significantly decrease lung metastasis in mouse breast cancer

Figure 7 summarizes the work model of miR-100. Taken together, miR-100 is highly expressed in TAMs and functions to promote TAMs phenotype via targeting mTOR pathway and upregulating IL-1ra’s secretion mediated by Stat5a. IL-1ra was then proven to enhance the stemness and chemo-resistance property of 4T1 tumor cells through Hedgehog pathway. Furthermore, inhibition of miR-100 can significantly decrease lung metastasis in 4T1 mouse breast cancer, and combination therapy with cisplatin shows enhanced effect. Therefore, our data reveal the crucial roles of miR-100 played in TME and its potential as a tumor therapeutic target.

## Discussion

In previous studies, miRNAs are described as “oncogenes” or “tumor suppressors” to illustrate their roles in cancer progress^27^. Whereas considering complicated cell composition in TME, the function of a single miRNA should be explored in a more comprehensive way. In this study, miR-100 represented the crucial role that a miRNA may play in TME.

The sequence of miR-100 is highly conserved between mouse and human, indicating that our research results may also have implications in humans. A large amount of evidence showed that miR-100 was downregulated in many human cancers including breast cancer, bladder cancer, NSLC, et al, in which miR-100 played important roles by regulating multiple cell processes, such as cell cycle, proliferation, differentiation, migration, invasion, and apoptosis^28^. Downregulation of miR-100 in breast cancer cells was reported to upregulate proliferation and survival-promoting oncogene insulin-like growth factor 2 (IGF2)^29^. Despite intensive research of miR-100 in cancer cells, there have been little studies in tumor-associated immune cells. Our study, for the first time, reported the function and regulation mechanisms of miR-100 in TAMs.

Moreover, in our study, macrophages that overexpressed miR-100 were capable of enhancing the invasion and chemotherapy resistance capability of 4T1 tumor cells. However, overexpression of miR-100 in 4T1 tumor cells decreased its ability of migration and invasion (data not shown), which was consistent with other reports^29^. Since one single molecule can function divergently in different cells and upon different stimulations, the opposite expression levels and functions of miR-100 in tumor cells and TAMs are comprehensible. In our animal experiment, inhibition of miR-100, which already expressed at a low level in tumor cells, did suppress tumor metastasis by rewiring the phenotype and function of TAMs.

Target genes of miR-100 in tumor cells have been intensively revealed. For instance, miR-100 was significantly upregulated in SK-BR-3 cells compared with other human breast cancer cells (MCF7, MDA-MB-453, T47D, HCC1954, and SUM149). Silencing miR-100 expression with anti-miRNA-100 oligonucleotide initiated apoptosis of SK-BR-3 cells by increasing the expression of MTMR3, which was also a target gene of miR-100 (ref.^30^). Meanwhile, FZD-8, a receptor of Wnt/β-catenin signaling pathway, was demonstrated a direct target of miR-100, and downregulation of miR-100 increased the migration and invasion of MCF-7 breast cancer cells^31^. miR-100 could also downregulate E-cadherin by targeting SMARCA5, a regulator of CDH1 promoter methylation^32^. Moreover, increasing miR-100 levels decreased the production of breast CSCs by attenuating the expression of the CSC regulatory genes SMARCA5, SMARCD1, and BMPR2^33^. In basal-like breast cancers, miR-100 was reported to inhibit maintenance and expansion of CSCs through Polo-like kinase1 downregulation^34^. According to the report that miR-100 could target mTOR in tumor cells^35^, our data confirmed that mTOR was also one of the target genes of miR-100 in macrophages.

Here our findings indicated a different mechanism of macrophage phenotype regulation conducted by mTOR. Previous work showed that TSC2 (mTOR negative regulator) knockdown could promote M2-function via enhancing mTOR/p-S6 pathway in human macrophages^36^, which is quite consistent with our data showing TSC1 (also mTOR negative regulator) K/O macrophages represented higher CD206 (TAMs marker) basal level than the control (Fig. 3d). Whereas in our study, mouse TAMs were proved to present both miR-100 higher and mTOR/p-S6 lower pattern when compared with “naïve” macrophages (Fig. 1d and Fig. 3a). MiR-100 could also promote CD206 expression by targeting mTOR/p-S6 pathway but failed to induce CD206 under the strong activation status of mTOR/p-S6 (Fig. 3c, d). All these data supported that the mTOR pathway was involved in the TAMs phenotype maintenance process mediated by miR-100, which demonstrated multiple roles of mTOR pathway in macrophage phenotype regulation network.

For the reason that there was a lack of data on transcriptional regulation of miR-100, some studies reported that miR-100 was commonly downregulated in human breast cancer due to hypermethylation of its host gene MIR100HG^32^. We treated macrophages with azacytidine, and miR-100 was upregulated (data not shown), which indicated that hypomethylation of the promoter of miR-100 may account for miR-100’s high expression in TAMs.

IL-1ra belongs to IL-1 family and it can block the effect of both IL-1a and IL-1b by competitively binding with IL-1 receptor^37^. It was reported that defect of IL-1ra correlated with IL-1-induced auto-immune disease^38^. In some clinical research, IL-1ra expression level and mutations were associated with increased risk of osteoporotic fractures^39^. In our study, macrophages secretion of IL-1ra was upregulated by overexpression of miR-100. We further explored the underlying mechanisms. We predicted the potential binding TFs on IL-1ra promoter region and then gained several candidates. Through siRNA knockdown and CHIP-PCR assay, we confirmed that Stat5a was the key TF that could bind to IL1ra promoter and upregulate IL-1ra expression. When moved to in vivo exploration, inhibition of miR-100 decreased the lung metastasis of 4T1 breast cancer, meanwhile, the level of IL-1ra in tumor tissue was also downregulated. The way that secretion of IL-1ra by TAMs influences tumor progression represented an important model of cell–cell interaction in TME.

It was also reported that IL-1 could promote carcinogenesis and metastasis by enhancing angiogenesis, endothelial cell activation, and lymphoid cell polarization^40^, and studies showed that targeting inflammasome/IL-1 pathway may provide a novel cancer immunotherapy^41^. IL-1ra (Anakinra), one of the anti-IL-1 strategies, has shown great potential in treating smoldering myeloma patients, with inhibition of its progression to frank neoplasia^42^. However, our study demonstrated the direct effect of IL-1ra on tumor cells, in the aspect of promoting tumor cells stemness and chemo-resistance, indicating that IL-1ra may function more than a cytokine. Moreover, a stemness-related pathway screen in 4T1 cells was performed in our study and it revealed that IL-1ra could upregulate Smo and Gli, receptor and TF of Hedgehog pathway, indicating the novel way of IL-1ra to impact cancer cell stemness. Our results also implicated IL-1ra’s ability to activate a pathway transduction by binding with an unreported receptor rather than IL-1 receptor.

Recently, targeted therapy has been one of the hotspots of cancer research, ranging from antibodies to small molecules delivered by nanoparticles. As TME is being extensively studied, TAMs have also attracted much more attention. With a lower chance of mutation and heterogeneity, TAMs are easier to be manipulated by medicines or other genomic methods, so that reversing or depleting TAMs has become a more promising strategy of targeted therapy. Blocking CSF1, the main factor to recruit and sustain TAMs, by antibodies or inhibitors, has been one of the most effective way to target and decrease TAMs, thus inhibiting cancer progression and metastasis^43^. Many clinical trials are currently evaluating efficacy of targeting CSF1/CSF1R signaling pathway as a remedy against multiple malignancies, including breast carcinoma, leukemia, and glioblastoma^44^. Since resistance to chemotherapeutic drugs of cancer cells was intensively reported, miRNAs were found to regulate breast cancer cell sensitivity to cisplatin including miR-302b^45^, miR-519d^46^, and miR-27a^47^. In our study, targeting miR-100 in TAMs could increase the chemo-sensitivity of cisplatin and inhibit lung metastasis of 4T1 breast cancer, indicating the clinical application potential of miR-100 inhibitor.

Here, we proved that miR-100 and IL-1ra played crucial roles in maintaining TAMs phenotype and promoting tumor progression, and inhibition of miR-100 with an antagomir could significantly suppress lung metastasis. Therefore, delivering miR-100 inhibitor by an antibody-linked nanoparticle will hold great potential as a therapeutic remedy. Given the fact that the IL-1ra^+^ population makes up the largest proportion of TAMs, targeting IL-1ra with an antibody or inhibitor will also be a method deserving research.

## Materials and methods

### Cell lines and reagents

Mouse breast cancer cell lines 4T1 and 4T1-luciferase and the mouse leukemic macrophage cell line RAW264.7 were obtained from Dr. R.A. Reisfeld (The Scripps Institute, La Jolla, CA, USA). The 4T1 and RAW264.7 cell lines were cultured in RPIM1640 medium (Hyclone, Cat. No. SH30809.01) supplemented with antibiotics (100 U/mL penicillin–0.1 mg/mL streptomycin) (Thermofisher Scientific, Waltham, MA, USA, Cat. No. 15240-062) and 10% fetal bovine serum (FBS) (Thermofisher Scientific, Cat. No. 1739463). Mouse PMs were obtained 3 days after the mice were intraperitoneally injected with 3% sodium thioglycolate (Millipore, Billerica, MA, USA, Cat. No. 1.08191.0500) and cultured in RPIM1640 medium supplemented with antibiotics (100 U/mL penicillin–0.1 mg/mL streptomycin) and 10% FBS.

### Animals

Female Balb/c mice, 6–8 weeks of age, were purchased from the Institute of Basic Medical Sciences of Peking Union Medical College. PMs from myeloid cell-specific TSC1 conditional knockout mice (LysMCreTSC1^loxp/loxp)^ and LysMCre-negative, TSC1^loxp/loxp^ littermates were donated from Hongbing Zhang (Peking Union Medical College, Beijing, China). All experiments involving the animals were performed according to the guidelines on laboratory animals and were approved by the Institute Research Ethics Committee of Peking Union Medical College.

### 4T1 mouse breast cancer model efficacy experiment

The 4T1 or 4T1-luciferase breast cancer cells (5 × 10^4^) were injected subcutaneously into the fourth mammary fat pad of each 8-week-old female mouse. Mice were randomly divided into each group (*n* = 5) based on their body weights and the order of tumor inoculation and there is no blinding. All mice were monitored daily. For the experiment shown in Fig. 2, miR-100 antagomir (chemically modified miRNA mimics) and negative control (Ribobio, Guangzhou, China) were injected intratumorally twice a week starting from day 7 post inoculation for 3 weeks. Luciferase activity was detected with Perkin Elmer IVIS weekly. On day 28 post inoculation, the tumor and lung of each mouse were harvested for flow cytometry, TAMs isolation, or immunohistochemical analysis. For the experiment shown in Fig. 6, miR-100 antagomir and negative control were injected via the tail intravenously every 4 days. Primary tumors were surgically excised from mice on day 14 post inoculation. All mice were euthanized on day 28, the lung tissues were separated, fixed in 10% neutral formalin for metastasis observation.

### TAMs isolation

The mouse breast tumor tissue (3 weeks) were collected from 4T1 mouse breast cancer model and mechanically dissociated with surgical scissors. Then the tumor tissues were digested with 0.05 g collagenase (Sigma, St. Louis, MO, USA, Cat. No. C-2674) in 15 mL HBSS (10% FBS) for 40 min in a 37 ℃ shaking incubator (190 rpm). After enzymatic dissociation, 15 mL HBSS (with 10% FBS) was added to stop the digestion. The tumor tissue suspensions were then strained through a 70-µm cell strainer (Corning, NY, USA, Cat. No. 352350), washed with PBS, and centrifuged at 1300 rpm, 4 ℃ (similar centrifugation parameters were used throughout the procedure). Red blood cells were lysed with ACK lysing buffer for 5 min followed by washing with MACS buffer (0.5% FBS in PBS, 10 mM EDTA, pH 7.2). The cell numbers were counted, and the suspension was again centrifuged; 90 µL MACS buffer (per 1 × 10^7^ cells) was used to resuspend the tumor cells and then 10 µL anti-CD11b microbeads (Miltenyi, Germany, Cat. No. 130-049-601) were added and incubated for 15 min at 4 ℃ in the dark. After washing with MACS buffer, TAMs were isolated using LS column (Miltenyi, Cat. No. 5130902344) in a magnetic separator.

### miRNA sequencing

The total RNA of TAMs was extracted by trizol reagent (Thermofisher Scientific, Cat. No. 15596018). The macrophages isolated from normal naïve mouse spleen served as control. miRNA sequencing was performed with illumina Hiseq 2500 (CapitalBio, Beijing, China). The raw data of the miRNA sequencing was further processed to make a category. The miRNA expression level was evaluated as TPM, different expressed miRNA was identified with cufflinks software. The miRNA sequencing data has been submitted to GEO repository and can be viewed at: https://www.ncbi.nlm.nih.gov/geo/query/acc.cgi?acc=GSE115295.

### qPCR

Small RNAs of RAW264.7 cells and PMs were purified and enriched with the miRNA Isolation Kit (Qiagen, Germany. Cat. No. 157046581). Reverse transcription was performed with TransScript First-Strand cDNA Synthesis Supermix (TransGene, Beijing, China, Cat. No. AT301-03). qPCR of miR-100 was performed with probes designed and synthesized by ABI (has-miR-100, 000437). The total RNA of RAW cells was extracted with trizol reagent. Reverse transcription was performed using TransScript First-Strand cDNA Synthesis Supermix, and real-time PCR was performed with TransScript Top Green qPCR supermix (TransGene, Cat. No. AQ-141-04) in triplicate.

### Western blot

RAW264.7 cells and PMs, transfected with miR100 mimic or inhibitor (Ribo Bio), were lysed with protein extraction reagent (Thermofisher Scientific, Cat. No. 78501). The cell lysate was incubated on ice for 30 min and centrifuged at 14,000 rpm for 10 min followed by the supernatant collection. Western blot was performed by using respective primary antibody (ILra: Abcam, Cambridge, MA, USA, Cat. No. ab-124962; Stat5a: Abcam, Cat. No. ab178941; Stat5b: Abcam, Cat. No. ab32043; mTOR: CST, Danvers, MA, USA, Cat. No. 2983; p-S6: CST, Cat. No. 4857; Gli1: Abcam, Cat. No. Ab151796; Gli2: ABclonal, Wuhan, China, Cat. No. A6510; Gli3: Abcam, Cat. No. Ab6050; Smo: ABclonal, Cat. No. A3274) diluted in PBS with 5% BSA. Then the blots were washed and incubated with appropriate secondary antibodies (anti-mouse IgG HRP: CST, Cat. No. 7076; anti-rabbit IgG HRP: CST, Cat. No. 7074). Quantitation of integrated pixel was analyzed by ImageJ.

### Flow cytometry

Macrophages or tumor tissue cells were treated with FcR blocker first and then stained with the F4/80-APC (Biolegend, San Diego, CA, USA, Cat. No. 123116) and CD206-FITC (Biolegend, Cat. No. 141704), 1:200 diluted in 100 µL PBS. Antibodies were incubated for 30 min at 4 ℃ in the dark. After washing with PBS twice, the cell suspensions were analyzed with flow cytometer (Millipore, Guava 5HT). The data were analyzed using FlowJo software (Tree Star Inc.).

### Small-RNAs transfection

Small-RNAs including nc mimic, miR-100 mimic, or siRNA were transiently transfected into macrophages using Lipofectamine RNAiMAX (Thermofisher Scientific, Cat. No. 1814773) according to manufacturer’s instructions.

### miRNA in situ hybridization and IHC staining

Pre-miR-100 probe and reagents for RNA scope were bought from ACD company (Advanced Cell Diagnostics, CA, USA) (BaseScope^TM^ Probe-BA-Hs-pre-MIR100, Cat. No. 709661; positive probe, Cat. No. 701041; negative probe, Cat. No. 701021; Detection reagent kit, Cat. No. 322910; pretreat 1&3 kit, Cat. No. 322381), and the pre-miR-100 in situ hybridization was performed in the human breast cancer tissue array (alenabio) according to manual book provided by ACD, which was followed with CD68 (Abcam, Cat. No. Ab955) IHC staining. Twenty cells in each tumor tissue slides within the tissue array were counted randomly, pre-miR-100 dots total number in 20 cells or CD68^+^ cells were recorded.

### ELISA

Tumor tissue lysates of 4T1 mouse breast cancer were homogenated and the supernatants were collected after centrifugation. The supernatants were then used for IL-1ra detection with Mouse IL-1ra/IL-1F3 Quantikine ELISA Kit following the protocol provided by R&D System (R&D, Minneapolis, MN, USA, Cat. No. DRA00B). Concentration of IL-1ra normalized to tissue weight was calculated according to the standard curve.

### Cell invasion assay

Transwell chambers (Corning Cat. No. 3422) with 8-µm-pore polycarbonate filters were used for assessing the invasion properties of tumor cells. 4T1 cells were seeded (1 × 10^5^ cells/chamber) on top of the cylindrical chambers in the presence of RPMI-1640 medium with 1% FBS. RAW264.7 cells (1 × 10^5^ cells/well) were seeded into the well bottom in a 24-well plate with 10% FBS culture medium. Cell invasion was observed after 24 h culture. Non-invasive cells were removed from the bottom of the chambers using cotton swabs. Invasive cells on the inner sides of the chamber were fixed in absolute methanol and stained with 0.1% crystal violet for 10 min at room temperature and subjected to microscopic observation.

### Chromatin immunoprecipitation and PCR

Chromatin immunoprecipitation (ChIP) was performed using a ChIP assay kit (Millipore, Cat. No. 17-295) to assess the binding of Stat5a to the IL-1ra promoter in RAW264.7 cells. RAW264.7 cells were collected, homogenized, cross-linked with formaldehyde (1%), and sonicated to produce sheared soluble chromatin. Precleared chromatin was incubated with Stat5a antibody (Abcam, Cat. No. ab178941) or IgG, as control, at 4 ℃ overnight. Immune complexes were collected with Protein A agarose beads. Chromatin complexes were eluted from the beads and cross-linking was reversed. DNA purified from the samples and input controls were analyzed for IL-1ra promoter sequences containing Stat5a putative response elements using quantitative PCR with the following primers: forward: 5′-CATTCTCTTGACAGGACTGAGCA-3′, reverse: 5′-CAGGTTTGAACTGAAGAGTTGTCC-3′; forward: 5′-CTCAGGTAATGGTCCTTGCCTT-3′, reverse: 5′-GCTGGCTCTGCATATACTGTGG-3′; forward: 5′-GCTGAATCATCTCTCTAGCTCCA-3′, reverse: 5′-GGAGTGACTTATGAGGGAGACTGA-3′.

### Cytokine array

The culture supernatant of RAW264.7 cells was collected 24 h after the transfection of miRNA mimics. Cytokines secreted by RAW264.7 cells, either transfected with miR-100 mimic or miR-negative control, were detected by using the Mouse Cytokine Array Panel A (R&D, Cat. No. ARY006) based on the Proteome Profiler Array Protocol. Cytokine array data were quantitated by using ImageJ software (http://rsbweb.nih.gov/ij/).

### Tumorsphere assay

4T1 cells were digested and counted, and 5000 cells were resuspended in 2 mL sphere-culturing medium (Stemcell, BC, Canada, Cat. No. 05611) and then seeded into 6-well ultra-low attachment plate (Corning, Cat. No. 16017027) for 5–7 days. The number of spheroids was counted after 7 days. Tumorsphere formation was monitored using an inverted Nikon microscope fitted with a camera.

### Signal pathway assays

Signal pathway assay was performed according to the protocol provided by the Cignal Finder Reporter Array kit (Qiagen, Cat. No. CCA-006L). 4T1 cells treated with IL-1ra at 100 ng/mL concentration for 6 h were harvested and then used for signal pathway array.

### Statistical analysis

Values were expressed as means ± s.e.m. Data were analyzed by using the Student’s *t*-test (two-sided). A value of *P* < 0.05 was used as the criterion for statistical significance; * indicated significant difference with *P* < 0.05, ** indicated significant difference with *P* < 0.01, *** indicated significant difference with *P* < 0.001.

## Supplementary information

Supplemental figure legends

supplemental figure1

supplemental figure2

supplemental figure3

supplemental figure4

supplemental figure5

supplemental figure6

Abbreviations list
