# Supplementary material for: miR-100 maintains phenotype of tumor-associated macrophages by targeting mTOR to promote tumor metastasis via Stat5a/IL-1ra pathway in mouse breast cancer
Source: Oncogenesis. 2018 Dec 19;7(12):97. doi: 10.1038/s41389-018-0106-y (PMC6299090; doi:10.1038/s41389-018-0106-y)
Supplement: Supplementary file 1 — Supplemental figure legends [file 41389_2018_106_MOESM1_ESM.docx]

**Supplementary figure 1.**

(A) Flow chart of 4T1 mouse breast cancer model establishment, tumor tissues were harvested after 3 weeks of injection. (B) Isolated macrophages purity and vitality were detected by flow cytometry, CD11b^+^7AAD^-^ subpopulation percentage was labeled. (C) TPM>5000 or TPM<5000 microRNA consisted black bar or gray bar, the percentage of microRNA quantity and reads among total microRNAs were shown. (D) Expression of pre-miR-100 was detected by RNA scope. Numbers of pre-miR-100-5p spots were counted from each cancer tissues or adjacent tissues.

**Supplementary figure 2.**

(A)Expression of MHC-I, MHC-II, CD80 and CD86 on miR-100 mimic transfected macrophages treated with gama-IFN were measured by flow cytometry, nc mimic as control group. (B) miR-100 or nc mimic transfected macrophages were treated with LPS (100ng/ml) and gama-IFN (50ng/ml) for 12 hours, and then incubated with Dil-labeled LDL for another 12 hours. Intracellular Dil signal was observed by a fluorescence microscope.

**Supplementary figure 3.**

(A) Mice body weight curve for either miR-100 or nc antigomir group. (B) miR-100 expression level was detected by using Taqman probe in all the two group mice, n=5, ***p<0.001. (C) and (D) Primary tumor weight or volume, n=5. (E) Lung to body weight ratio in groups of mice injected with miR-100 antigomir or nc antigomir , n=5. (F) CD206 mRNA expression in TAMs from two group mice primary tumor tissue was detected by qPCR, n=5, **p<0.005.

**Supplementary figure 4.**

(A) and (B) Quantitation results of Fig.3A and Fig.3B. R stands for RAW264.7 cell in nc-mimic-R, and P stands for PM in nc-mimic-P (C) Peritoneal macrophages isolated from wild type and TSC1 myeloid-specific knockout mouse were transfected with miR-100 mimic or nc mimic for 24 hours, CD206 mRNA expression was detected by qPCR.

**Supplementary figure 5.**

(A) Schematic diagram of TFs screening with two gene pools. (B) Candidate TFs information and predicted binding sites number. (C) mRNA knockdown efficiency of candidate TFs siRNA was validated by qPCR. (D) Quantitation results of Fig.4F. (E) Quantitation results of Fig.4G.

**Supplementary figure 6.**

(A) Quantitation results of Fig.5G. (B)Animal experiment scheme for miR-100 and cisplatin combination therapy. (C) Primary tumor growth curve of negative control group and 3 therapeutic groups. （D）Picture of lung tissue showing metastatic nodes with red arrow. n=5
