# Supplementary figures and images for: miR-100 maintains phenotype of tumor-associated macrophages by targeting mTOR to promote tumor metastasis via Stat5a/IL-1ra pathway in mouse breast cancer
Source: Oncogenesis. 2018 Dec 19;7(12):97. doi: 10.1038/s41389-018-0106-y (PMC6299090; doi:10.1038/s41389-018-0106-y)

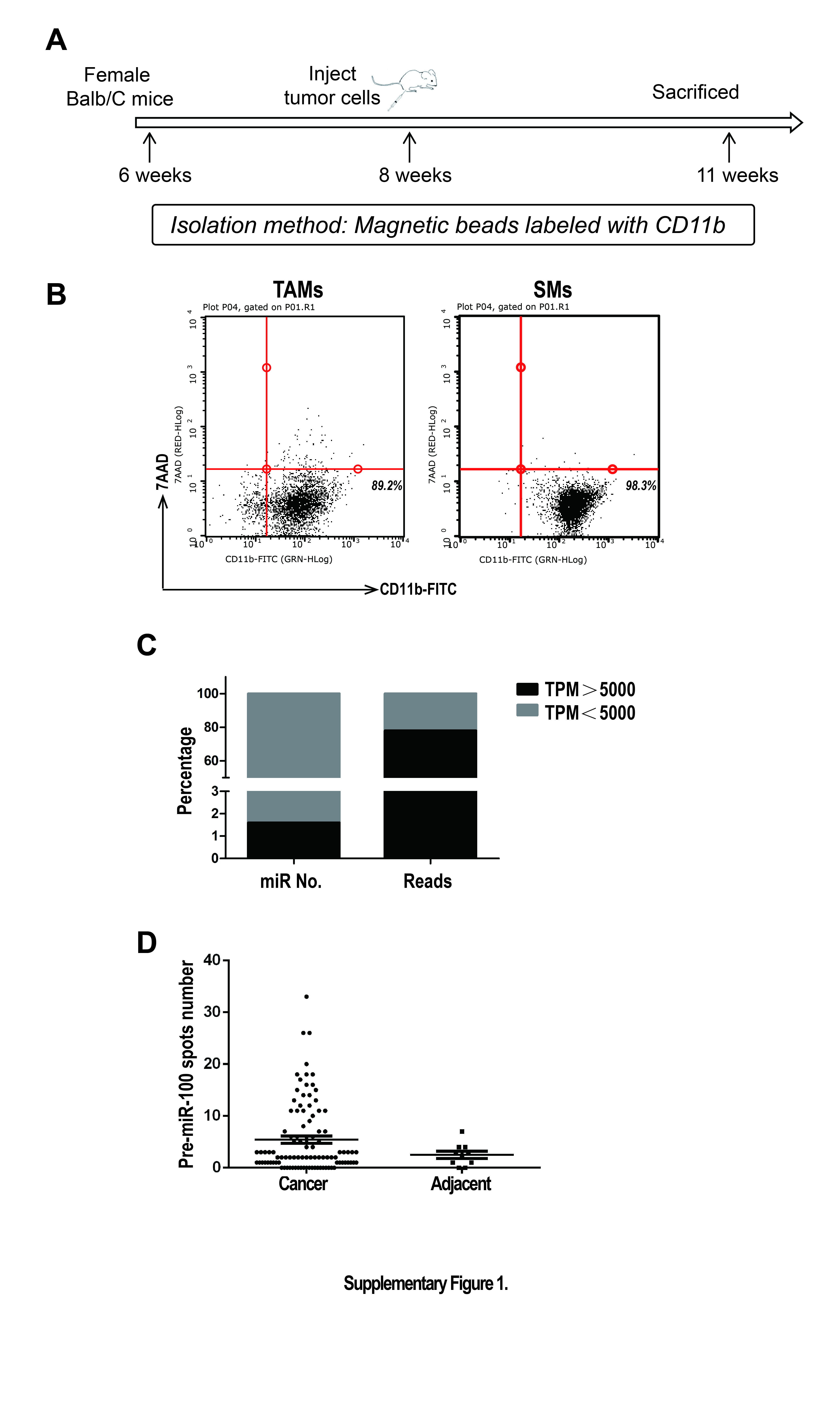

Supplement: Supplementary file 2 — supplemental figure1 [file 41389_2018_106_MOESM2_ESM.jpg]

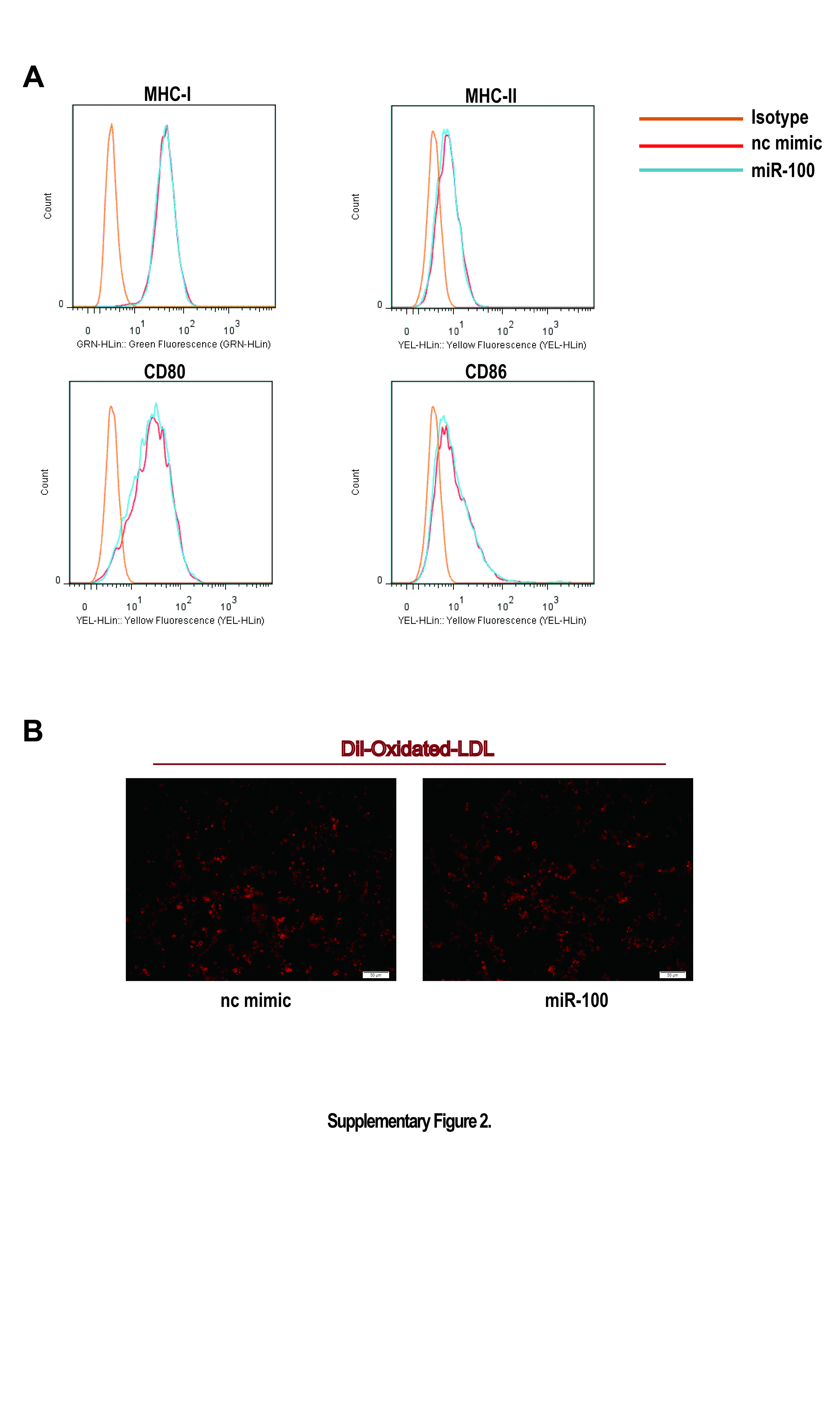

Supplement: Supplementary file 3 — supplemental figure2 [file 41389_2018_106_MOESM3_ESM.jpg]

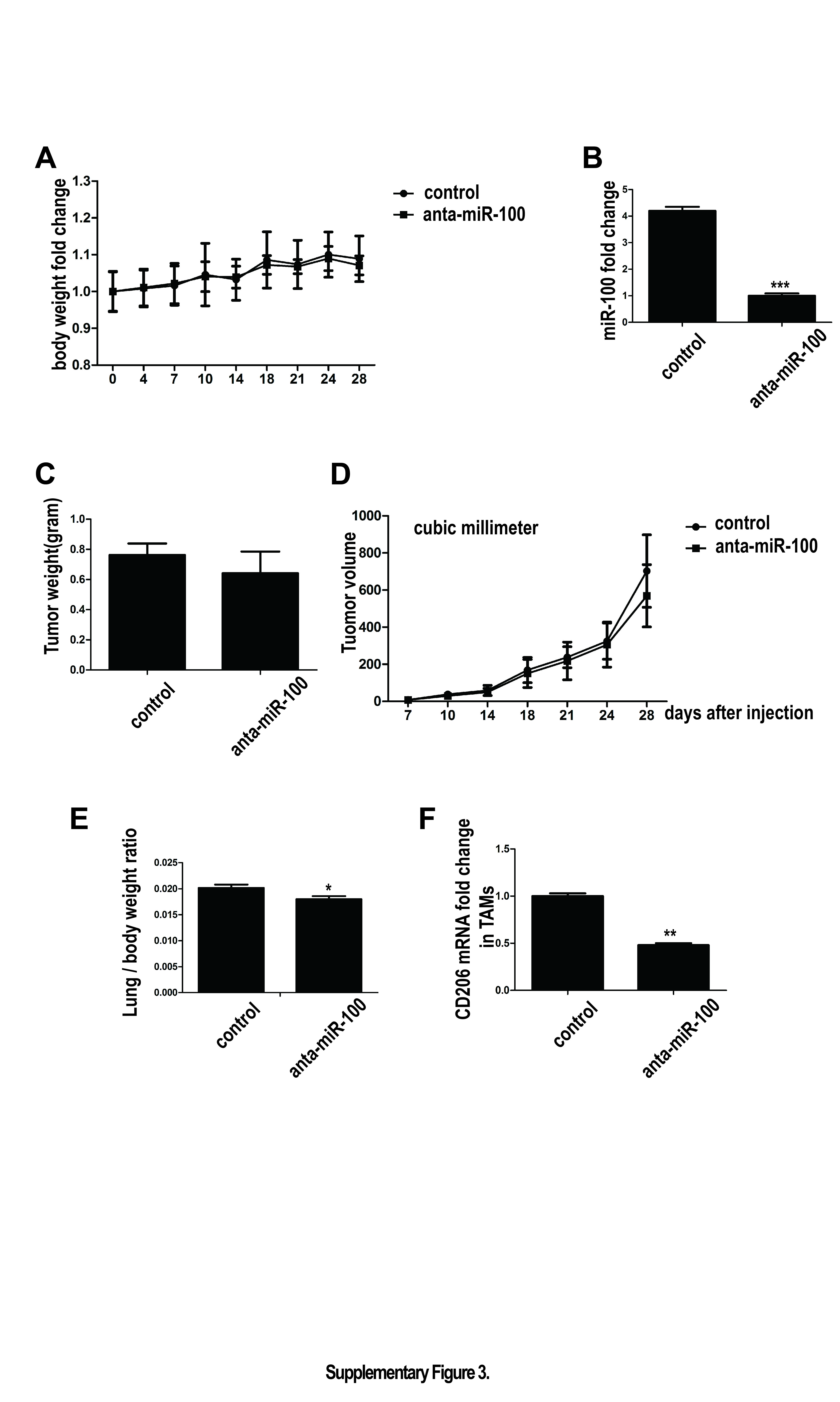

Supplement: Supplementary file 4 — supplemental figure3 [file 41389_2018_106_MOESM4_ESM.jpg]

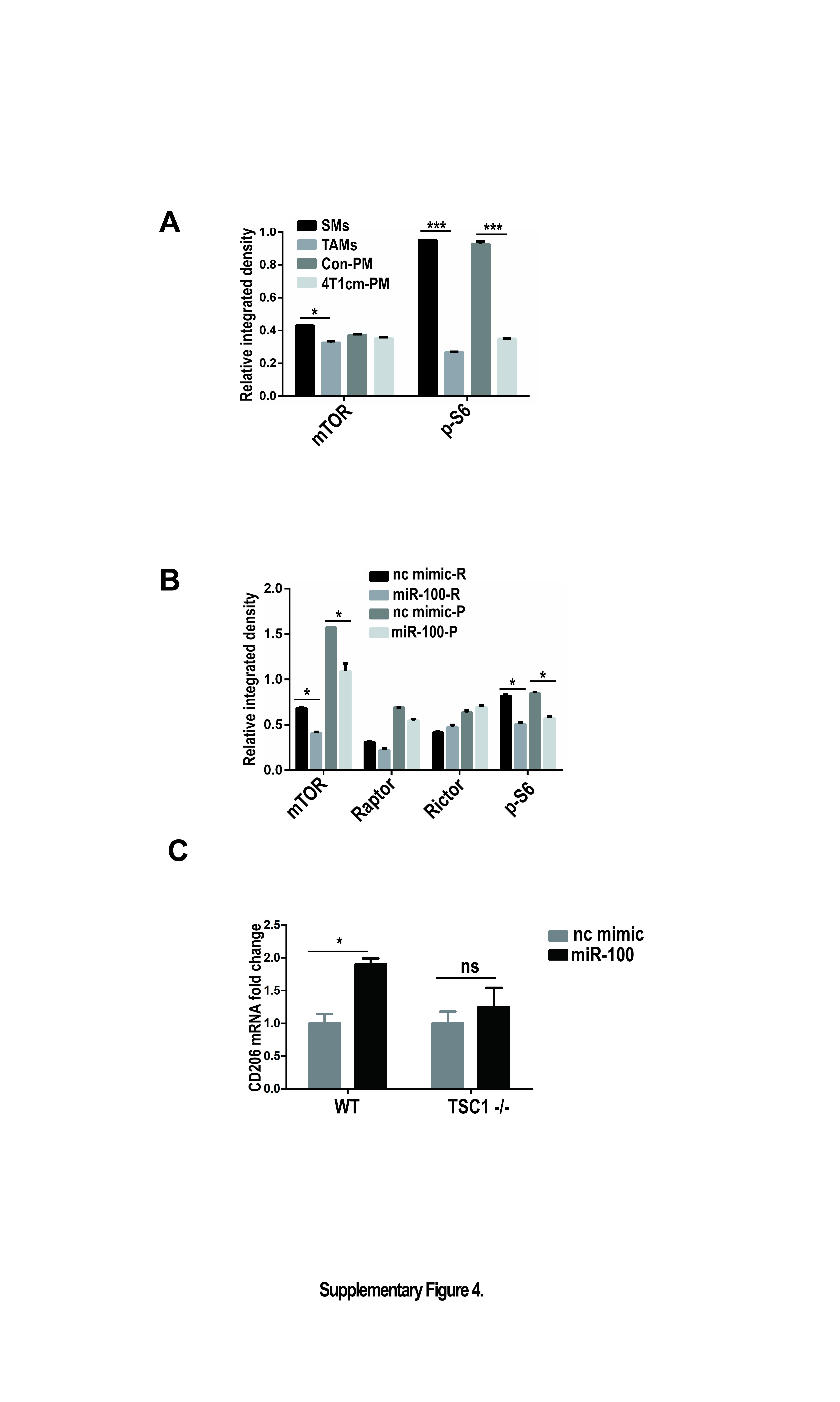

Supplement: Supplementary file 5 — supplemental figure4 [file 41389_2018_106_MOESM5_ESM.jpg]

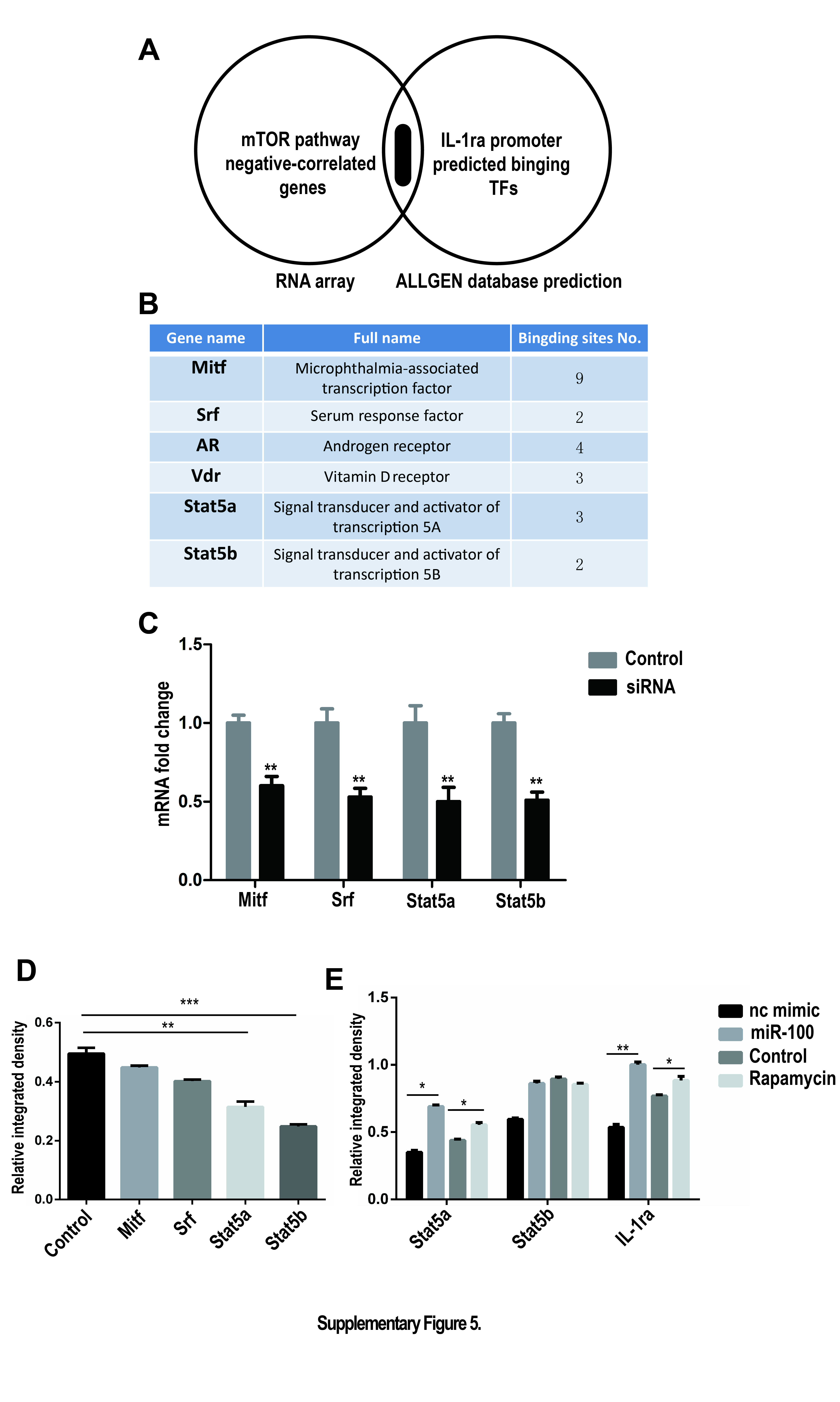

Supplement: Supplementary file 6 — supplemental figure5 [file 41389_2018_106_MOESM6_ESM.jpg]

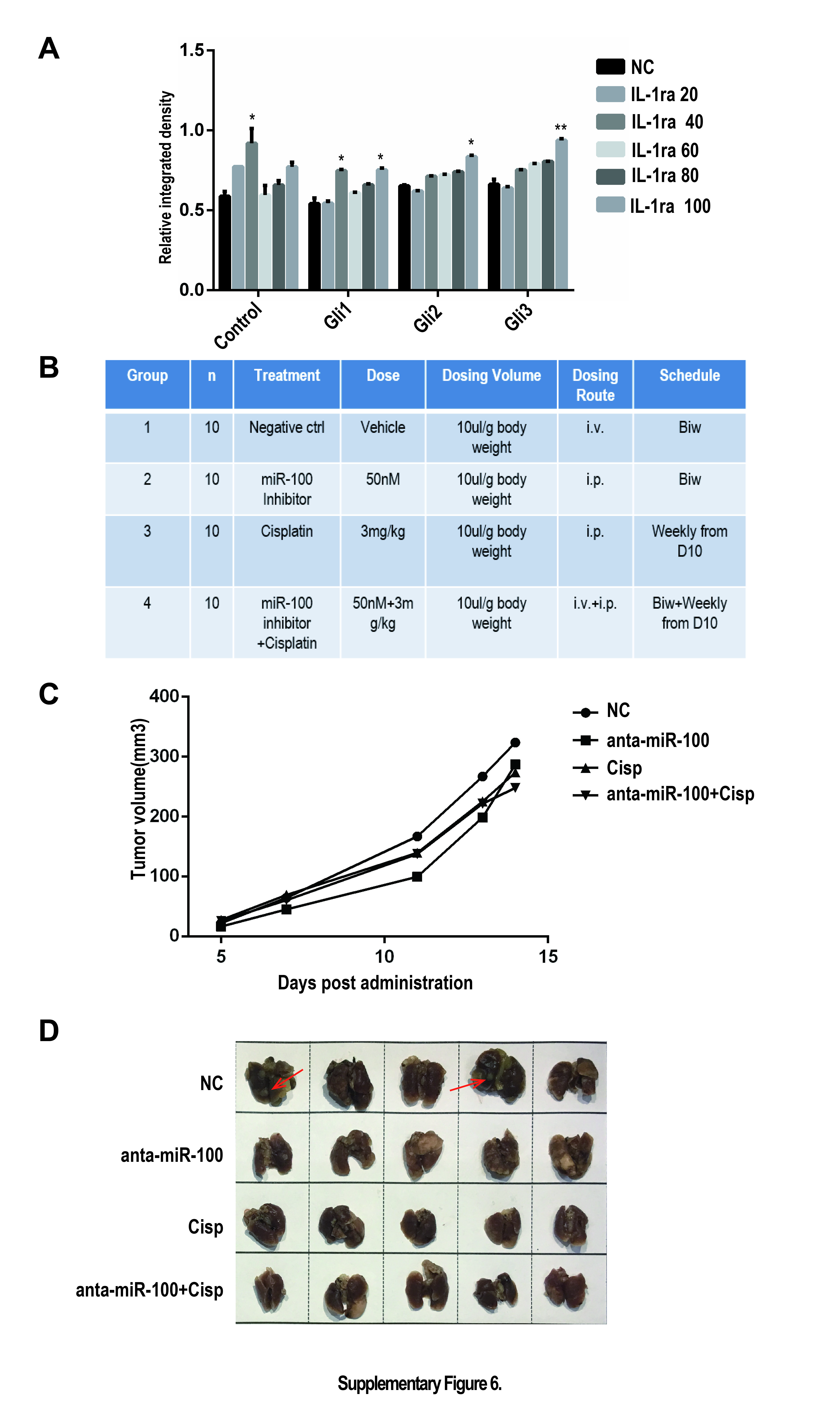

Supplement: Supplementary file 7 — supplemental figure6 [file 41389_2018_106_MOESM7_ESM.jpg]
