# Supplementary material for: miR-100 maintains phenotype of tumor-associated macrophages by targeting mTOR to promote tumor metastasis via Stat5a/IL-1ra pathway in mouse breast cancer
Source: Oncogenesis. 2018 Dec 19;7(12):97. doi: 10.1038/s41389-018-0106-y (PMC6299090; doi:10.1038/s41389-018-0106-y)
Supplement: Supplementary file 8 — Abbreviations list [file 41389_2018_106_MOESM8_ESM.docx]

| Abbreviations list | |
| --- | --- |
| BMPR2 | Bone morphogenetic protein receptor type-2 |
| CD206 | Clusters of differentiation206 |
| CD68 | Clusters of differentiation68 |
| CDH1 | Cadherin 1 |
| ChIP-PCR | Chromatin immunological precipation-Polymerase chain reaction |
| CSCs | Cancer stem cells |
| CSF1 | Colony stimulate factor 1 |
| CSF1R | Colony stimulate factor1 receptor |
| ELISA | Enzyme linked immunosorbent assay |
| EMT | Epithelial |
| FZD-8 | Frizzled-8 |
| Gli1 | Glioma-associated oncogene homolog 1 |
| Gli2 | Glioma-associated oncogene homolog 2 |
| Gli3 | Glioma-associated oncogene homolog 3 |
| HE | Hematoxylin-eosin |
| HOXA1 | Homeobox protein hox-A1 |
| IGF2 | Insulin like growth factor-2 |
| IL | Interleukin |
| IL-1ra | Interleukin-1 receptor antagonist |
| IRAK1 | Interleukin 1 receptor associated kinase 1 |
| LPS | Lipopolysaccharide |
| miRNAs | MicroRNAs |
| MTMR3 | Myotubularin related protein 3 |
| mTOR | Mamalian target of rapamycin |
| NF-κB | Nuclear factor κB |
| NSLC | Non-small cell lung cancer |
| O/E | Overexpression |
| PMs | Peritoneal macrophages |
| p-S6 | Phospho-s6 |
| Ptch | Patched homolog 1 |
| qPCR | Quantitative polymerase chain reaction |
| SMARCA5 | SWI/SNF-related matrix-associated actin-dependent regulator of chromatin subfamily A member 5 |
| SMARCD1 | SWI/SNF-related matrix-associated actin-dependent regulator of chromatin subfamily D member 1 |
| Smo | GPCR-like protein Smoothened |
| SMs | Spleen macrophages |
| Stat5a | Signal transducer and activator of transcription 5a |
| Stat5b | Signal transducer and activator of transcription 5b |
| TAMs | Tumor-associated macrophages |
| TFs | Transcriptional factors |
| TGF | Transforming growth factor |
| TME | Tumor microenvironment |
| TNF | Tumor necrosis factor |
| TPM | Transcripts per million |
| TRAF6 | TNF receptor associated factor 6 |
| TSC1 | Tuberous sclerosis 1 |
